# Being a refugee in childhood and health in adulthood

**DOI:** 10.1093/eurpub/ckac131.526

**Published:** 2022-10-25

**Authors:** E Mattelin, AR Khanolkar, L Korhonen, F Froberg

**Affiliations:** 1 Barnafrid, Linköpings University, Linköping, Sweden; 2 Center for Social and Affective Neuroscience, Linköpings University, Linköping, Sweden; 3 School of Life Course and Population Sciences, King’s College London, London, UK; 4 Department of Child and Adolescent Psychiatry, Linköping Univerisity, Linköping, Sweden; 5 Department of Global Public Health, Karolinska Institutet, Stockholm, Sweden

## Abstract

**Aim:**

To examine whether individuals who migrated to Sweden as refugees in childhood are more likely to experience poor mental and general health and violence in adulthood, compared to individuals born in Sweden, or who were migrants but not refugees.

**Methods:**

This study included 151,614 individuals who answered the Swedish National Public Health Survey in 2018 or 2020. We grouped the participants into refugees in childhood, migrants in childhood, or non-migrants. Information about outcomes - mental health, general health, and risk behaviors - was collected through questionnaires. We analyzed the associations using logistic regressions.

**Results:**

Overall, having been a refugee in childhood was generally not associated with poor general health and mental ill-health, risk behaviors, or exposure to violence, compared to those who were migrants or born in Sweden. However, there were some exceptions. For example, young men, who were refugees in childhood, had a higher likelihood of suicide attempts than non-migrants. Interestingly, childhood refugees and childhood migrants were less likely to use drugs and to have risky alcohol use as adults, compared to non-migrants, but were more likely to be risk gamblers.

**Conclusions:**

Being a refugee in childhood was not, in general, associated with negative health consequences in adulthood with some exceptions, such as gambling, that will be discussed in the presentation.

**Key messages:**

• In this study, refugee experience in childhood is not, in general, associated with worse health outcomes in adulthood.

• There are certain risk groups that need to be highlighted such as young males with refugee experience in childhood.

